# Non-invasive continuous-time glucose monitoring system using a chipless printable sensor based on split ring microwave resonators

**DOI:** 10.1038/s41598-020-69547-1

**Published:** 2020-07-31

**Authors:** Masoud Baghelani, Zahra Abbasi, Mojgan Daneshmand, Peter E. Light

**Affiliations:** 1 Microwave to Millimeter Wave (M2M) Lab., Department of Electrical and Computer Engineering, University of Alberta, Edmonton, AB Canada; 2 Alberta Diabetes Institute and the Department of Pharmacology, University of Alberta, Edmonton, AB Canada; 3 Department of Electrical Engineering, Ilam University, Ilam, Iran

**Keywords:** Biomedical engineering, Electrical and electronic engineering, Diagnostic markers, Sensors and probes

## Abstract

This paper reports a highly sensitive, non-invasive sensor for real-time glucose monitoring from interstitial fluid. The structure is comprised of a chip-less tag sensor which may be taped over the patient’s skin and a reader, that can be embedded in a smartwatch. The tag sensor is energized through the established electromagnetic coupling between the tag and the reader and its frequency response is reflected on the spectrum of the reader in the same manner. The tag sensor consumes zero power as there is no requirement for any active readout or communication circuitry on the tag side. When measuring changes in glucose concentrations within saline replicating interstitial fluid, the sensor was able to detect glucose with an accuracy of ~ 1 mM/l over a physiological range of glucose concentrations with 38 kHz of the resonance frequency shift. This high sensitivity is attained as a result of the proposed new design and extended field concentration on the tag. The impact of some of the possible interferences on the response of the sensor’s performance was also investigated. Variations in electrolyte concentrations within the test samples have a negligible effect on the response of the sensor unless these variations are supra-physiologically large.

## Introduction

The World Health Organization estimates that there are > 500 M people worldwide who have diabetes^1^. Diabetes is primarily characterized by poorly controlled blood glucose concentrations that, if allowed to remain chronically high (hyperglycemia), result in the development of serious and life-threatening diseases such as stroke, heart attack, heart failure, kidney failure, adult blindness and amputation^2^. Moreover, many patients also experience episodes of very low blood glucose (hypoglycemia) that can rapidly lead to coma and death^3^. The most common glucose-sensing technology in use today are finger-prick based glucose strips, although this requires sampling many times per day and the continual purchase of the consumable once-use strips. As such, patient compliance to regular glucose monitoring is often not possible. Moreover, real-time continuous glucose monitoring offers a much more accurate assessment of the large fluctuations in blood glucose that can occur in patients with diabetes^4,5^. In this regard, newer glucose-sensing technologies include the placement of a thin needle-like sensor under the skin to measure glucose levels within interstitial fluid that closely tracks with blood glucose levels^6^. Although this technology removes the need for finger-prick blood sampling and can sample glucose levels every few minutes, the sensor is consumable, is expensive, and requires the insertion of a new sensor every 10–14 days^7^. Therefore, many patients cannot take advantage of this technology. What is really required is a non-invasive, reliable and cost-effective technology that measures glucose in real-time^8^.

To date, there has been much interest in developing novel glucose-sensors and a variety of sensor technologies have been tested. One of the most promising therapeutic systems for diabetes patients is the artificial pancreas, whereby an automated insulin delivery system is coupled to a real-time glucose sensing devise^9,10^. Such technology promises much tighter control of blood glucose levels. However, the main drawback associated with the artificial pancreas is the real-time and continuous glucose monitoring system. Indeed, a great deal of research has focused on the development of optimal real-time non-invasive glucose-sensing^11–15^, that optical, transdermal and infra-red techniques. The majority of techniques are based on near infra-red spectroscopy (NIRS) and impedance spectroscopy^16,17^. While, other optical techniques have also been tested that include Raman spectroscopy^18^, tomography^19^ and photoacoustic methods^20^. Optical methods suffer from absorption by other materials, low signal to noise ratio (SNR), thermal noise, calibration drift, environmental susceptibility, power consumption and cost. Transdermal glucose monitoring systems are based on glucose extraction from interstitial fluid (ISF) using reverse iontophoresis techniques^21,22^ followed by a chemical sensor for glucose concentration measurement. Although they claim to be non-invasive, they actually inject electrical current through the skin. Furthermore, the sensors degrade over time and the technology is expensive. The thermal emission infra-red thermography technique which uses the body temperature for gaining an understanding about blood glucose levels^23^ is expensive and inaccurate with respect to glucose monitoring. Other techniques such as saliva^24^, breath^25^, sweat^26–28^ and tear analysis^29^ are being developed but all have inherent problems and limitations.

Due to their high-quality factor, low cost, and non-invasive nature, microwave resonator-based sensors have garnered a great deal of interest in the last decade. Their potential applications have now expanded from the oil and gas industry^30,31^ to microfluidic and biomedical^32–34^ and to other industrial applications^35–37^ that include glucose-sensing^38–45^. Since blood glucose is tightly regulated, even the largest variations that occur in diabetes are within a relatively small range (2–25 mM/l). To date, much research has been published that report non-physiological values for glucose sensing in the 1,000 mM/l, emphasizing low sensitivity as the major problem associated with existing microwave-based resonator sensor technology. Sensitivity for this kind of sensors is defined as the resonance frequency variation versus the change in the permittivity of the material under the test (MUT).

**Figure 1:**
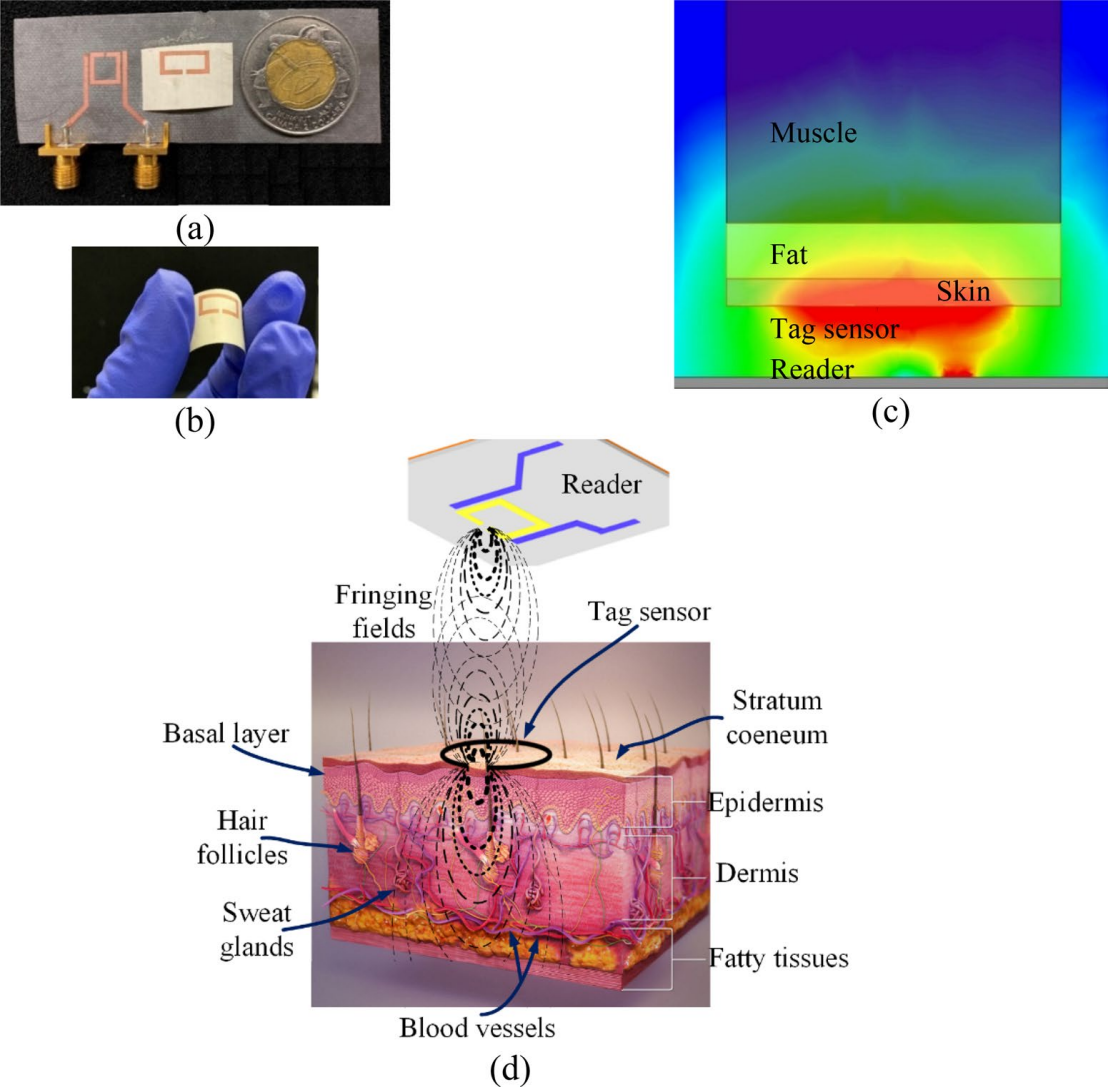
Conceptual representation of the operation of the proposed glucose monitoring system. (**a**) Fabricated sensor system including the reader and the tag, (**b**) sensing tag flexibility (**c**) electromagnetic simulation of the sensor over the external tissues from skin to muscle. It could be seen that the electromagnetic field concentration is much higher at skin region and into the fat with decay of the field at deeper levels such as muscle in muscles. That is the main reason behind using ISF as it is an ideal fluid for glucose monitoring because the higher field concentration results in the higher sensitivity of the system to the variation in the permittivity (this image is obtained from HFSS simulation and edited in MS Word). (**d**) Detailed illustration depicting the proposed application of this technology. Note that the field concentration decays with increased distance from the sensor. The immediate layer in contact with the sensor, Stratum Corneum, contains no ISF and hence doesn’t contribute to the glucose monitoring response (i.e. frequency variation). The next epidermal layer, basal layers, contains around 40% of ISF and according to its low distance from the sensor, it is the dominant layer determining the sensor response. id. Considering the cells as static variables, which is a reasonable assumption because of their very slow dynamics, the most important variables in this layer that could interfere with the response of the propose sensor are dehydration and saline variation. These topics are experimentally studied throughout the paper to have negligible impact on the sensor response. The overall epidermal thickness is about 100 µm varies depending to the location. The next layer is dermis containing around 40% of ISF, less cells, very small percentage of lymph fluid and about 8% of blood plasma. Again, the main possible interfering parameters in this layer are the same as epidermis. Since the average dermal thickness is about 2 mm, the layers after dermis have negligible impact on the sensor response because the field strength at those layers is very low. In addition, this figure conceptually presents the communication between the tag sensor and the reader which is mostly accomplished through fringing fields (the biological part of this part is from an open source document available at https://commons.wikimedia.org/wiki/File:3D_medical_animation_skin_layers.jpg and edited in MS Visio).

Herein, we present a microwave resonator-based sensor with a novel design and new structure that has been developed for the specific requirements of glucose measurement from interstitial fluid. Figure 1 presents the fabricated glucose sensing system including the reader and the tag as well as the conceptual structure of the sensor and its application as a glucose monitoring system. The sensor is constructed of a substrate-less split ring resonator tag operating as the sensing element that is electromagnetically coupled with a traditional split ring resonator designed at a different frequency as the reader. Since the sensing part is without substrate, huge improvement in sensitivity is achieved as discussed in “Results and discussion”. This sensitivity achievable is much greater than the values for glucose reported in the literature to date. The structure enables the integration of the reader and the required circuitry within a smart watch, cell phone or an application specific device for distance measurement capabilities. The sensing element itself is just a metallic trace which could be simply taped on the patient’s skin and is replaceable at extremely low cost. In addition, since the power is coupled from the reader to the sensor through fringing fields, the tag itself consumes zero power negating the requirement for a power supply on the sensor itself and its required energy is provided from the reader through electromagnetic coupling.

**Figure 2:**
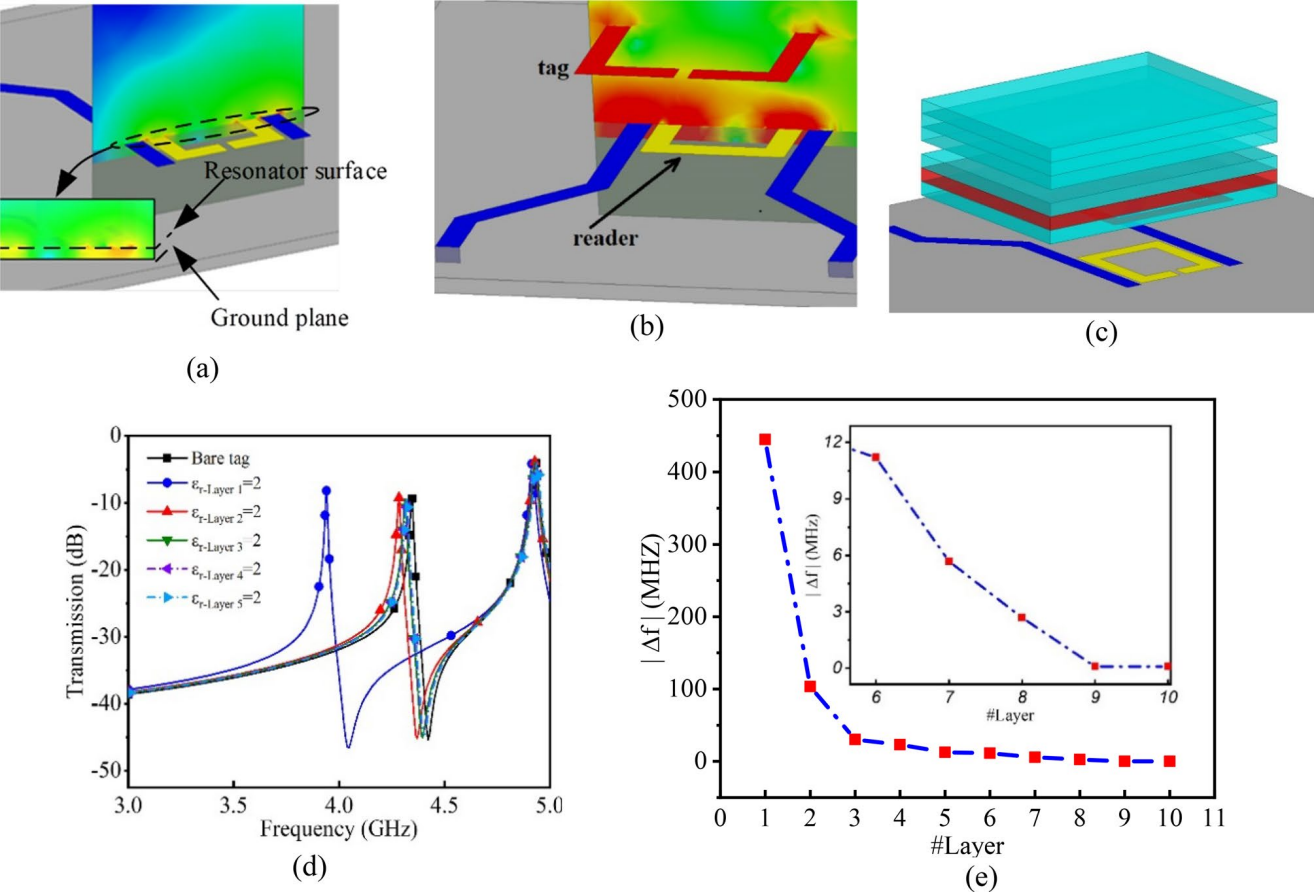
(**a**) The field concentration of a traditional microwave ring resonator; note that the field is concentrated between the resonator and its ground-plane and therefore the substrate plays the most important role in determining of the resonance frequency. Also, the field concentration at the region above the sensor, which usually considered as the sensing area is smaller which limits the sensitivity of microwave resonator-based sensors. (**b**) Perspective view and field concentration of the resonator, it could be seen that the fields around the tag is almost constantly distributed in both on top (where material-under the test is placed) and underneath (where could be considered as its substrate). Therefore, one could expect higher frequency dependency of the tag to the MUT which could be translated to higher sensitivity in comparison with traditional microwave resonator sensors. (**c**) The simulation setup for studying the effect of the distant of the MUT from the tag on the sensitivity of the sensor. In this simulations, ten layers with the same thickness of 1 mm above the tag have been tested with the same permittivity as the air and only the permittivity of one of them has been changed to 2 at each step determined by layer number in parts (**d**) and (**e**) [images in parts (**a**), (**b**), and (**c**) are obtained from HFSS]. (**d**) S21 spectrum of some of the simulation results. (**e**) frequency variations versus the layer number (i.e. distance), it could be seen that, as the distance becomes higher, the sensitivity decreases in an exponential manner and therefore variations in the permittivity of MUT at higher distances could negligible in comparison with the same variations at lower distances, therefore we expect the sensor to measure glucose concentration variation in ISF rather than blood, so all the experiments are designed based on ISF measurement and the variations of its components.

The proposed sensor is capable of measuring glucose concentration in the range of 0–200 mM/l with the precision of ~ 1 mM/l which should provide sufficient sensitivity for the accurate real-time measurement of interstitial fluid glucose levels from patients with diabetes. Our results demonstrate that microwave bio-sensing technology can be optimized to reproducibly detect glucose concentrations ranging from 2–25 mM/l in physiological solutions designed to mimic interstitial fluid. Importantly, this range is the same as the blood glucose levels seen during hypoglycaemia and hyperglycemia in diabetes patients. Furthermore, the current design is also compatible with the development of wearable electronics applications.

## Results and discussion

In this section the proposed glucose monitoring sensor is presented alongside with the schematics, design approach, featured parameters, characteristics, analysis, and various accomplished experiments for glucose concentration measurement in different conditions as well as an intense discussion including analysis of different parameters effects on the measurement.

### Chipless tag resonator sensor design

Figure 2 presents the perspective view of the field concentrations of the chipless microwave sensor for glucose sensing applications. The sensor is a ring-shaped copper trace designed to work around 4 GHz, as shown in Fig. 2. This frequency is selected because there is a considerable difference between water, as the main material in interstitial fluid, and saturated glucose solution permittivity while their loss factors are still small, and therefore measuring at this frequency will result in a significant frequency shift and hence the device sensitivity^46^. Also, since loss factor at this frequency is still low for water, the quality factor of the resonator will remain high which is of high significance for high precision measurements. Since the sensor is constructed of two resonators, there are two peaks and notches in the spectrum. In this measurement, only the notch related to the tag will be considered. As shown in Fig. 2, the sensor contemplates the variations in the medium introduced to the tag which is skin and its underneath including interstitial fluid and blood depending on the sensor mounting location. Variations in the materials permittivity in the regions subjected to higher concentration fields has more contribution to frequency shift. For quantifying this fact, in Fig. 2, an MUT with different layers stacked above the sensor is presented. All the layers have the same dielectric permittivity of 1 and the same thickness of 1 mm and only permittivity of one of them is changed to 2 at each step. Results illustrated in Fig. 2c verifies our justifications. Based on this observation, it seems glucose concentration variations in ISF has much more impact on frequency shift of the sensor than its variations in blood. Therefore, in the succeeding subsections only fluids and components of ISF are modeled.

### Detection mechanism

In this part, different parameters utilized as the outputs or detection mechanisms of the presented sensor for glucose monitoring are described. Also, some high frequency simulations and analysis will be provided verifying supremacy of the performance of the proposed sensor.

#### Frequency variation

The resonance frequency of the microwave split ring resonators (*f*_*r*_) is a function of inverse effective permittivity (*ε*_*r,eff*_) of the resonator’s environment^47^ which is generally an unknown function of the substrate dielectric permittivity and the permittivity of the experimental setup and MUT as well.

1 $${f}_{r}\propto \frac{1}{\sqrt{{\varepsilon }_{r,eff}}}.$$

When MUT is introduced to a resonator, the overall effective permittivity of the system is changed and therefore the resonance frequency of the resonator. This shift in the resonance frequency is therefore a measure for determining of the introduced material for a constant volume. Frequency shift measurement is a robust parameter against additive noise and also is easy to measure. Readout circuitry have been developed with the detection limits in the range of 100 ppb (parts per billion) easily which makes high resolution frequency shift measurement both precise and straightforward^48^.

#### Amplitude variation

Another output of microwave resonator which could be invaluable for attaining an insight into MUT is amplitude variation. Amplitude variation is mostly occurred as the result of variations in conductivity of MUT^49^. This usually happens when concentration of electrolytes changes inside ISF. Since the conductivity spectrum of materials differs in trend (if not completely orthogonal) from their permittivity, studying amplitude variations could be very useful.

#### Sensitivity analysis

**Figure 3:**
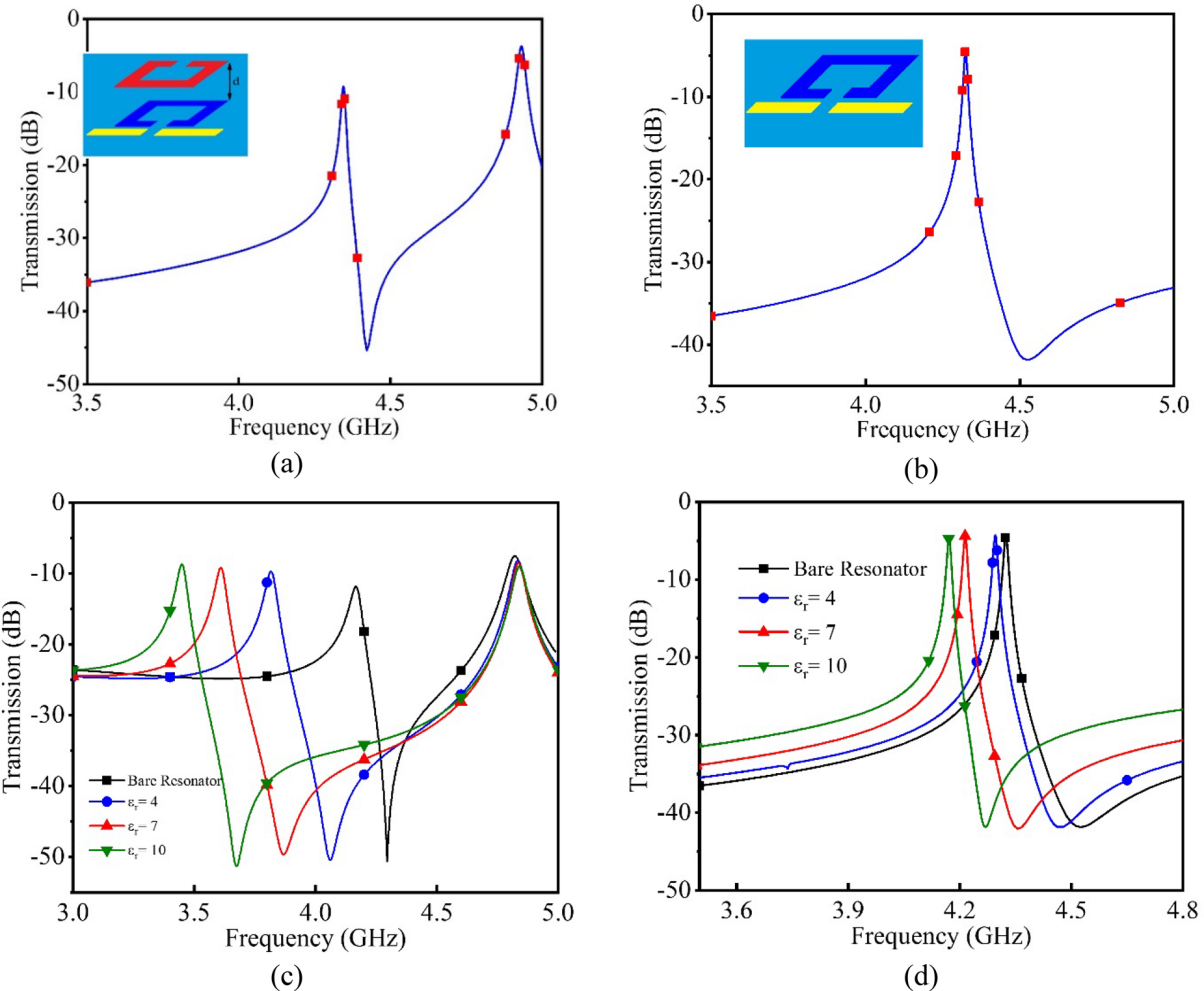
Sensitivity comparison between the presented sensor and traditional microwave resonator sensors. (**a**) Proposed sensor sensitivity test setup with a superficial material with the relative permittivity between 1 (bare resonator) and 10. (**b**) Traditional microwave resonator sensor with the same volume and permittivity. (**c**) and (**d**) The spectrum of both setups from sections (**a**) and (**b**) respectively as well as their resulted spectrums from MUT relative permittivity variations from εr = 1 to εr = 10. It could be seen that, frequency shift related to the proposed sensor is 700 MHz (**c**) in comparison with 200 MHz for the traditional sensor (**d**) under the same condition.

**Figure 4:**
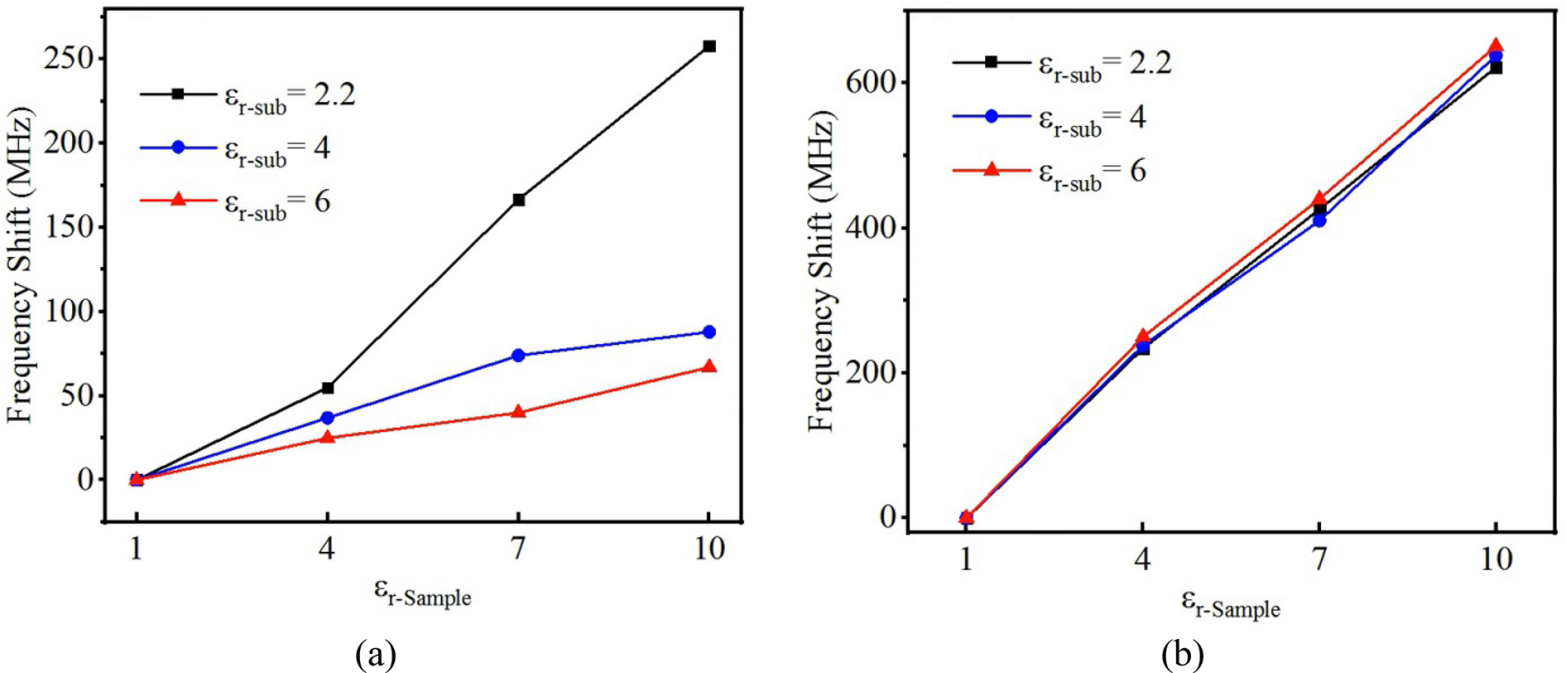
Comparison between the impact of substrate in determining the resonance frequency of the traditional and proposed sensors. Frequency shift versus MUT permittivity for different permittivity values for substrate for (**a**) traditional sensors, (**b**) proposed sensors; it could be seen that the effect of substrate permittivity in traditional resonator sensors is dominant while its impact is negligible for the proposed sensor. This is the reason of higher achieved sensitivity of this design in comparison with the traditional sensors.

Considering frequency shift as the main output parameter for the sensor, sensitivity could be defined as the frequency shift versus permittivity variations of MUT for a certain volume. Since, each research uses arbitrary container volume and shape, for having a meaningful understanding of sensitivity improvement in the proposed sensor, a comparison between traditional microwave resonators and the current introduced sensor designed at the same frequency is presented here. As illustrated in Fig. 3, a superficial material with specific volume and shape covering the whole area of both resonators with ε_r_ = 4 is introduced as MUT. The frequency shift resulted from relative permittivity variation to 10 for the proposed sensor is 700 MHz which is more than 3.5 times higher than the frequency shift for the traditional resonator. Limited sensitivity of the traditional resonator is as the result of confined electromagnetic fields between the resonator and its ground plane (see Fig. 2a). In traditional resonators, because of this phenomenon, substrate has a more important role in defining the resonance frequency rather than MUT. Because of the removing of the substrate for the tag in the presented work, the main variable parameter defining the resonance frequency of the tag is the MUT permittivity. For studying this concept, another simulation has been accomplished for both conventional and presented resonators. As depicted in Fig. 4, different substrate permittivity has been used with different permittivity for MUT for both traditional and the proposed sensors. It could be seen that, for traditional resonator sensors, substrate permittivity is the dominant parameter in determining the resonant frequency of the structure while the impact of substrate permittivity variations on the proposed sensor is very small and even negligible. For the remaining of this paper, we define sensitivity as the frequency variation resulted from 1 mM/l of glucose concentration change for a specific test setup.

#### Distant measurement analysis

**Figure 5:**
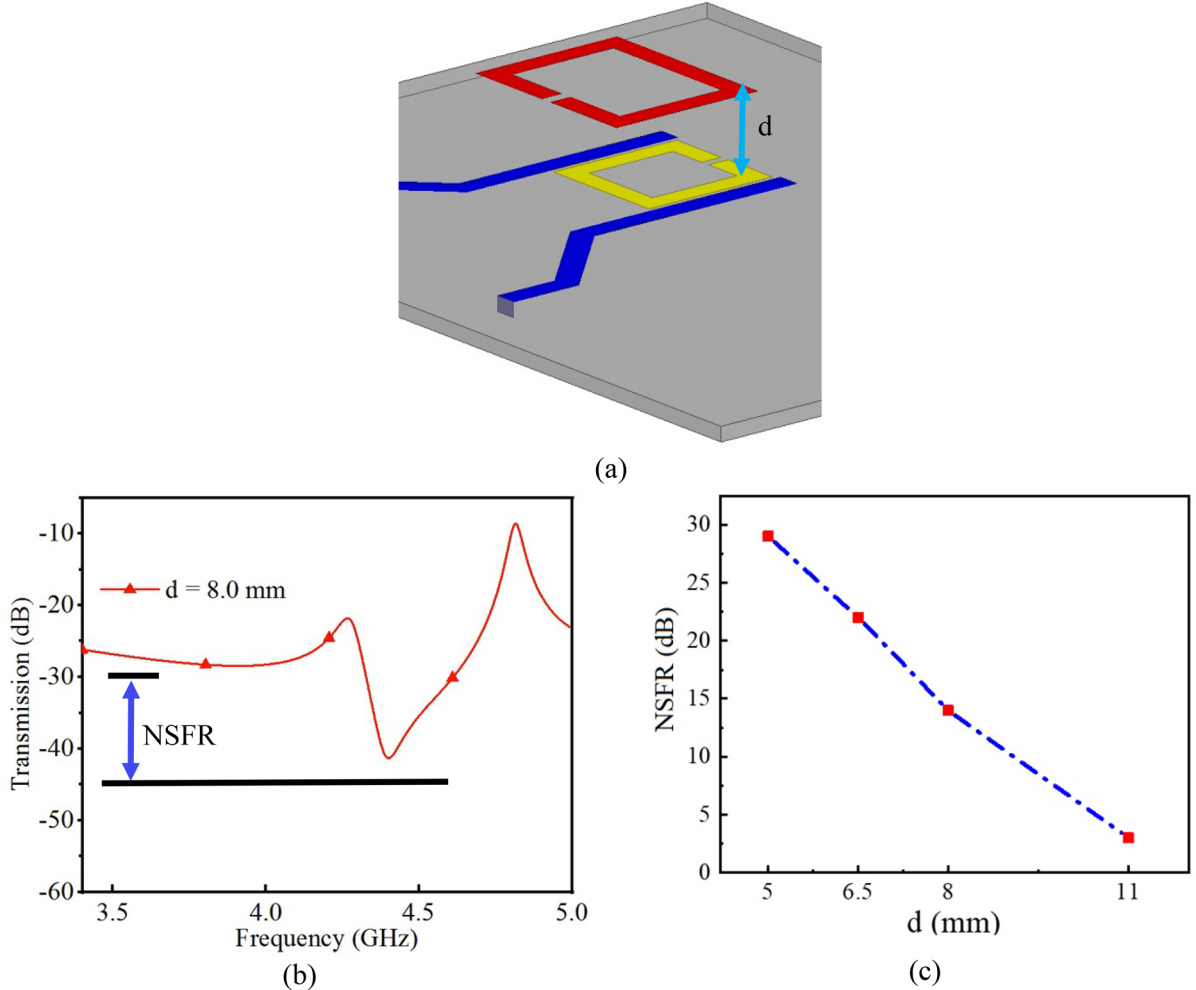
(**a**) Simulation setup for characterization of distance measurement of the proposed sensor (image is obtained from HFSS). (**b**) Definition of the notch to signal floor ratio (NSFR) for the presented simulation. (**c**) NSFR of the signal versus the distance of the sensor from the reader.

Another notable feature of the presented work is the distant sensing capability. This characteristic is especially important for wearable electronic applications. In addition to capability of embedding the reader in a smart watch, phone or a gadget, this remarkable feature brings up new paramount benefits such as zero power consumption, extremely low cost, and small size for the sensing tag. For having a better insight into this characteristic, another simulation has been accomplished by placing MUT with specific relative permittivity on top of the tag and increasing the distance between the reader and the tag. It could be seen in Fig. 5 that tag continue to communicate with the reader for almost 11 mm with absolutely zero power which is completely enough for our application.

### Experiments

**Figure 6:**
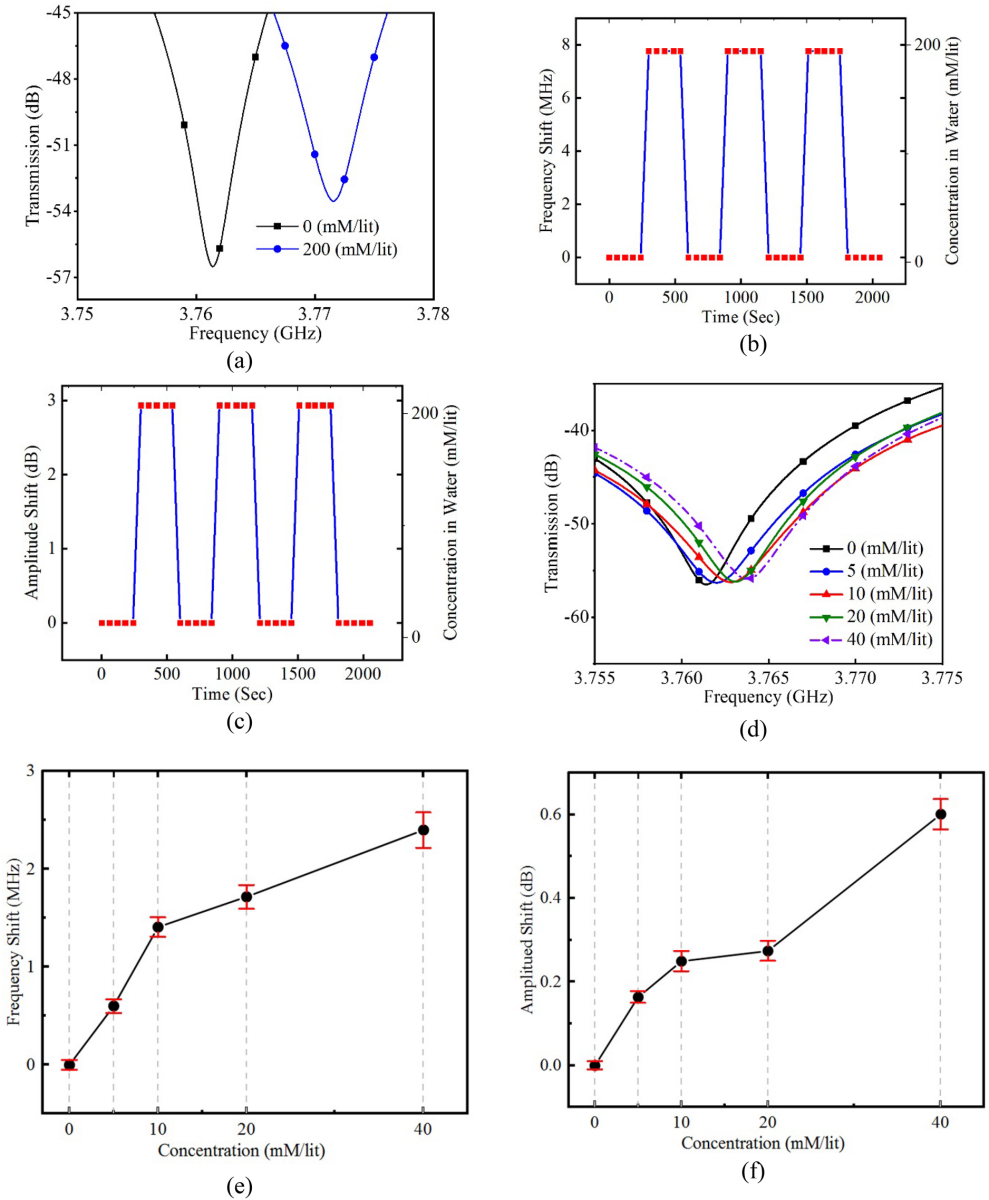
(**a**) S21 experimental response of the sensor for extreme case of introducing samples with 0 mM/l and 200 mM/l of glucose concentration fo the sensor. (**b**) Frequency shift versus glucose concentration for the extreme case of 0 and 200 mM/l glucose concentration in DI water. It could be seen that the response of the sensor is very consistent and repeatable. (**c**) Amplitude shift versus glucose concentration for the extreme case of 0 and 200 mM/l glucose concentration in DI water. (**d**) S21 response of the sensor for small variations of glucose concentration in DI water from 0 to 40 mM/l. (**e**) Frequency shift versus glucose concentration for concentration variations from 0 to 40 mM/l. It could be seen that great results have been achieved with very high average sensitivity of 60 kHz/1 mM/l of glucose concentration. (**f**) Amplitude shift versus glucose concentration for concentration variations from 0 to 40 mM/l.

Various measurements have been accomplished verifying the performance of the proposed non-invasive glucose measurement sensor. First of all, glucose concentration measurement in deionized (DI) water is carried out. For studying consistency and stability of the sensor as well as setup a return-to zero test is accomplished with as high concentrations of glucose as 200 mM/l (Fig. 6). Although this value is unrealistically high, but it will provide invaluable insight through consistency of the sensor performance by introducing DI water with zero glucose concentration and DI water with 200 mM/l glucose concentration alternatively to the sensor. Figure 6d sketches the resonance frequency notch amplitude of S21 response of the sensor. It could be seen that the sensor response is both stable and repeatable. Also, high sensitivity characteristic of the sensor is noticeable. To the best of our knowledge, the achieved sensitivity of this work, 60 kHz/1 mM/l of glucose concentration which is superior to the best results reported in literature regardless of shape and volume of MUT. This means, the response of the sensor is less susceptible to environmental noises than its conventional counterparts.

**Figure 7:**
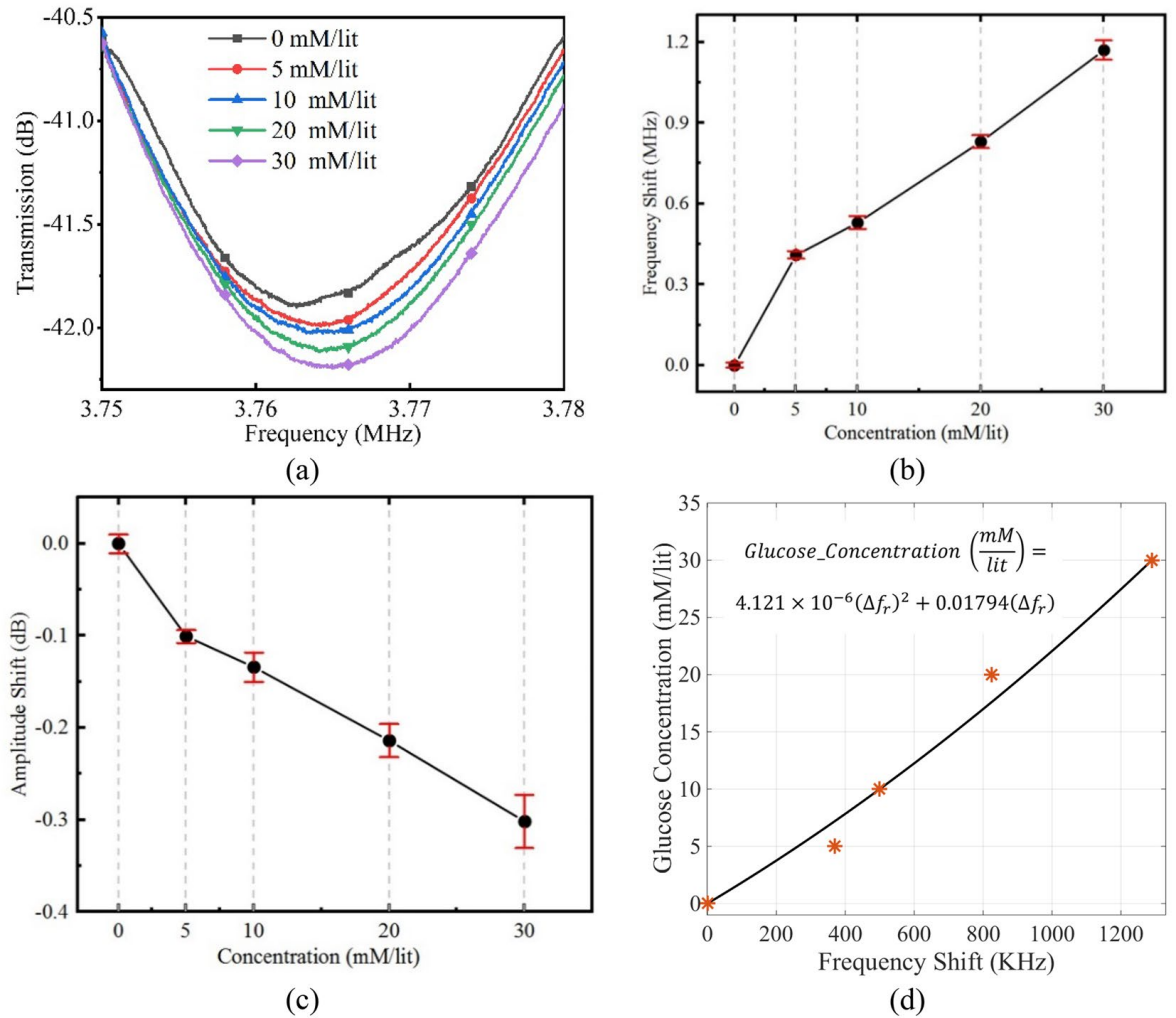
Experimental results of samples with glucose concentration in DI water with 10% of horse serum content. (**a**) S21 response of the sensor for glucose concentrations from 0 to 30 mM/l. (**b**) Amplitude variations versus glucose concentration from the same experiment. (**c**) Frequency shift versus glucose concentration. It could be seen that, according to lower permittivity of serum in comparison with water, the total permittivity of water-serum solution is reduced and therefore the impact of the glucose variation on the overall permittivity of the solution is reduced as well which results in a lower sensitivity of 43 kHz/1 mM/l of glucose concentration. (if we had return to zero results we could integrate them with this fig as well), (**d**) a calibration curve for glucose concentration versus the measured frequency shift. Note that the calibration curve provides a reasonable fit with the data point despite of some errors which may be related to slight variability in the experimental samples.

For the next step, samples are prepared with 10 volumetric percent of horse serum for modelling ISF. Both return-to zero and small variations of glucose concentration samples have been tested with promising results achieved as sketched in Fig. 7. For achieving a better idea on the performance of the sensor, it is common to address the glucose concentration versus frequency shift as the measured data. An interpolation curve fitting process then accomplished based on the resulting data. These results are presented in Fig. 7d.

**Figure 8:**
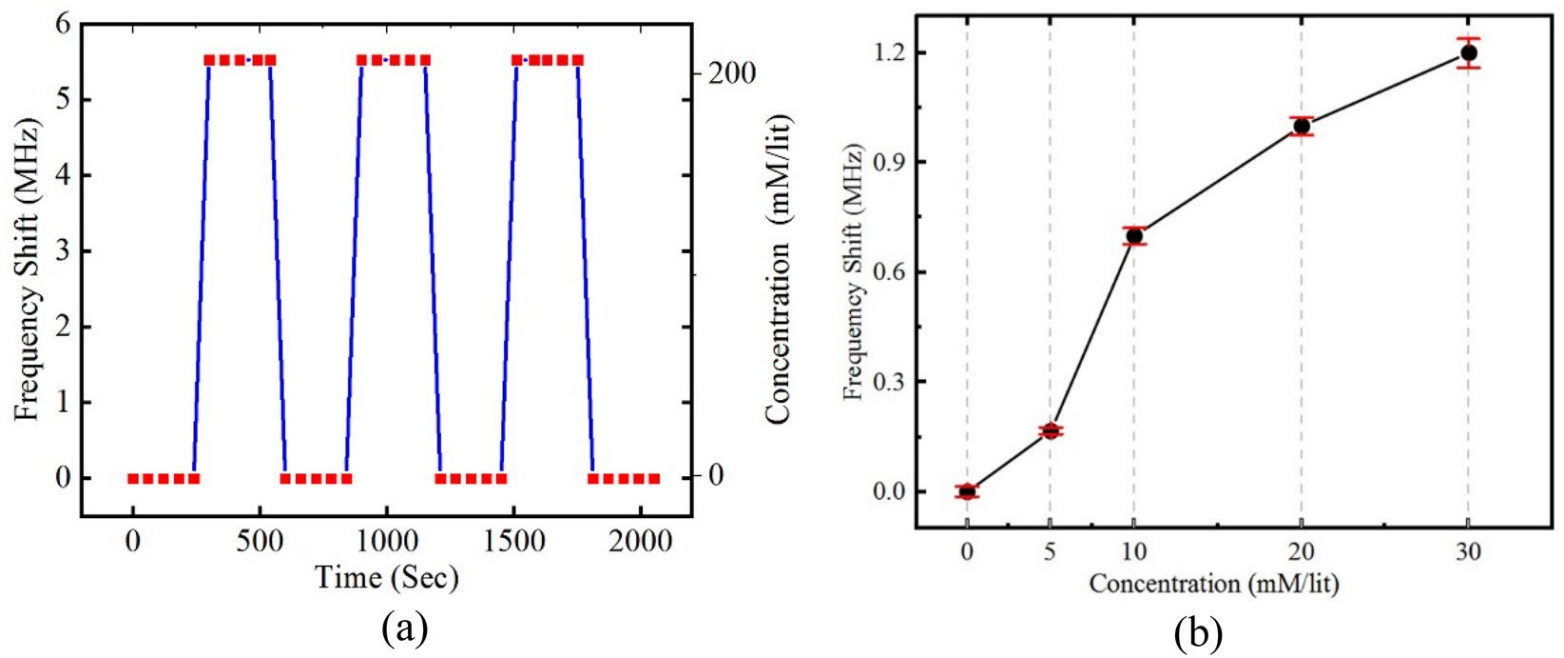
Experimental results of impact of glucose concentration variation in samples with DI water + serum + saline solution. (**a**) Frequency shift of the sensor as the response to alternatively changing the glucose concentration from zero to 200 mM/l. It could be seen that, the proposed sensor presents a stable and repeatable response over time. (**b**) Frequency shift of the sensor as the response of small variation of glucose concentration. It could be seen that, according to introducing of the skin between the sensor and the sample, the overall sensitivity is reduced to 38 kHz/1 mM/l of glucose concentration variation.

To further mimic a more physiological condition, we performed glucose sensing experiments through a layer of mouse skin. In these experiments, saline is included in the sample with electrolytes and ionic concentrations described in "Results and discussion" section. According to conductivity increasing of the samples, the amplitude of the notch frequency is increased. For this experiment, a shaved mice skin with about 300 µm thickness wrapped inside a sealed plastic bag is used between the sensor and the liquid. Hence, the sample is located in further distance from the sensor. As illustrated in Fig. 8, the sensitivity of the sensor is decreased with the same justification as Fig. 2 as the result of increasing the distance between ISF sample and the sensor. However, the sensitivity of the system to changes in glucose concentration is still superior to other non-invasive technologies published to date.

### Discussion

Although microwave resonators possess impressive characteristics, there is still a very challenging issue remained. Since any variation in the permittivity of MUT is reflected in frequency shift of the resonator, there is a concern about the uncertainty of the actual source of frequency shift. For addressing this issue, an extensive discussion part including some experiments is provided.

**Figure 9:**
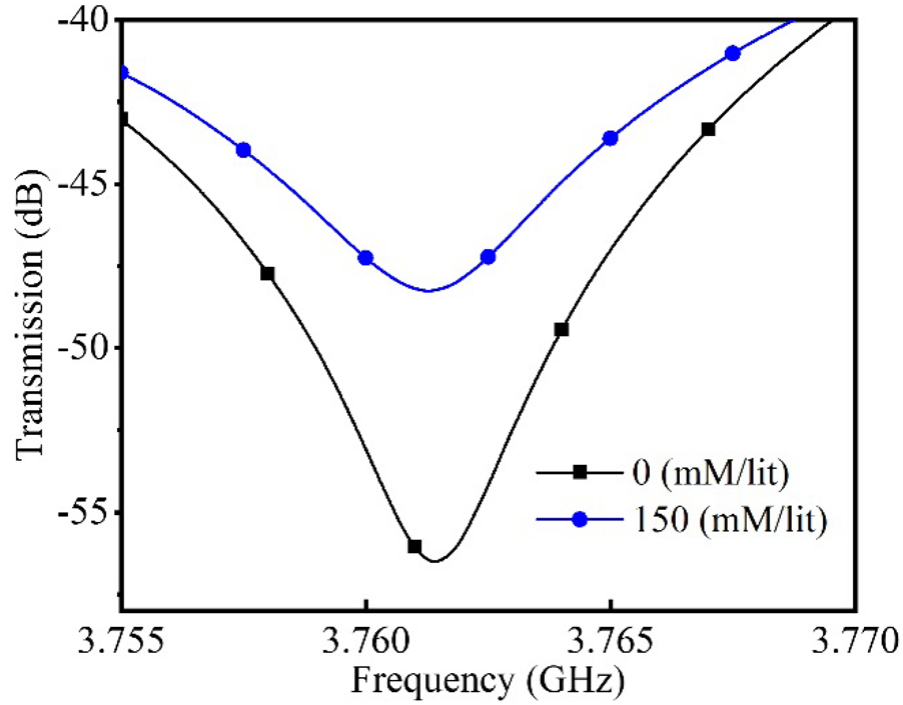
Effect of saline variations on the response of the sensor; here only Na and Cl concentrations have been changed as the major electrolytes in ISF from 0 to 150 mM/l. Although the maximum variation happens in human body is limited from 136–150 mM/l, an exaggerate variation is tested here to presents the proof of concept. It could be seen that saline concentration has in important impact on the amplitude of the response but its resulting frequency shift is less than 20 KHz which is completely negligible. The case would be even more negligible in real life case, because of less variations in the electrolytes.

The presented sensor aims to measure glucose concentration in ISF which is a fluid contains around 40% of human body’s water surrounding the cells acting as the nutrient transporting from blood capillaries and waste collecting medium for the cells. Beside water and plasma, ISF also contains glucose, fatty acids and salts. So far, glucose variation effects have been tested. Here, we provide some experiments for studying the effects of mineral variations on the frequency shift of the sensor. The main ions in ISF are, sodium, potassium, chloride, calcium, magnesium, bicarbonate and phosphate. Since sodium and chloride ions have one or more orders of magnitude higher variation range in comparison with the other ions, for the sake of simplicity, they are considered as the only variable ions the experiments. It could be seen from Fig. 9 that since ions mostly affect the conductivity of the MUT, it won’t change the frequency of the sensor. Hence, since frequency change is considered as the main output of the sensor, ionic concentration variations is unlikely to not interfere with results from glucose related frequency shift. In addition, fatty acid concentration variation inside ISF is in the range of < 1 mM/l and therefore its effects are minimal on the frequency shift in comparison with effect of glucose variation.

**Figure 10:**
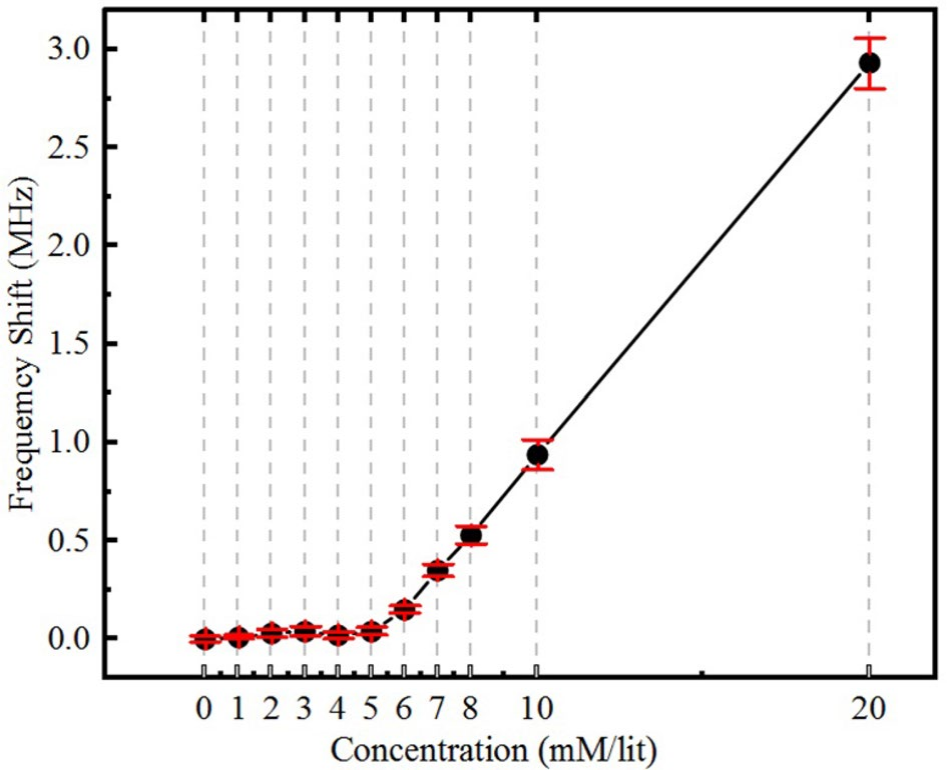
Frequency shift as the results of dehydration. It could be seen that, low to moderate dehydration (up to 5%), have very small interference with the response of the sensor. But, severe dehydration could have the same impact on the frequency shift as about 50 mM/l variation in glucose concentration. Although it’s results in huge error, severe dehydration is a deadly problem and patients should be hospitalized immediately accordingly. So, one could consider the effect of low to moderate dehydration as a minimal error which is less than the impact of 0.3 mM/l variation in glucose concentration.

Another important parameter to consider is ionic concentration changes that manifest as a result of hydration levels. For example, mild dehydration often occurs regularly in humans. Dehydration directly affects the water content in ISF and therefore could change its permittivity and consequently affects the performance and precision of the sensor. Sample preparation method is presented in the next section. Figure 10 presents the frequency shift versus dehydration percentage with all the other variables remaining constant. Our results demonstrate that low to moderate dehydration has a minor effect on the frequency shift even less than the effect of 1 mM/l variation in glucose concentration. However, severe dehydration has the potential to interfere with the frequency shift resulting from glucose variations and therefore compromise the glucose sensitivity of the sensor. Therefore, further development of this sensor technology will have to consider the impact of severe dehydration on sensor accuracy. The real-time applicability of the sensor is achieved because of instant variation in glucose concentration in the MUT results in its dielectric permittivity which changes the effective permittivity of the sensor’s environment and consequently results in frequency shift (see Eq. 1).

A comprehensive comparison between the presented structure and some of the state-of-the art works using methods other than microwave is outlined in Table 1. Another quantitative comparison between different microwave techniques-based glucose sensors and the current paper is presented in Table 2. Although, some of the summarized works seems to have higher sensitivity than the proposed work, but those are mostly as the result of lower distances between their resonators and sample due to using of extra-thin microfluidic channels. This justification is completely in agreement with the concept presented in Fig. 2. It could be seen from Fig. 2e that the frequency shift (i.e. sensitivity) is drastically reduced with increasing the distance of the sample from the sensor in an exponential manner. We present the design and testing of non-invasive glucose sensor with a very high sensitivity despite the considerable distance between the sensor and the testing medium that would be expected in real-life biosensing applications.

**Table 1:** Comprehensive comparison between other flexible real-time glucose monitoring systems and the proposed sensor.

References	Sensing technique	Focused material	Sampling method	Sensor substrate	Comments
^21^	Electrochemical sensor	ISF	Transdermal ISF extraction using reverse-iontophoresis process	Flexible PET sheet	Flexible and wearable structure Semi-invasive The target fluid is available upon the reverse iontophoresis extraction process High saturability
^50^	Resistance and capacitance variations of an electronic device	Tear	Tear is available	500 nm thick Parylene	Wearable but hard to implant Semi-invasive Complex structure Target fluid availability is high No-saturability
^51^	Electrochemical sensor	ISF	Transdermal extraction and collection of ISF using an extraction chip	Flexible polyimide substrate	Flexible and wearable structure Semi-invasive based on ISF extraction chip Availability of the target fluid is low for the sensor High-saturability
^52^	Electrochemical sensor	Sweat	Sweat collection using an ultrathin and stretchable design	Flexible polyimide substrate	Flexible and wearable structure Non-invasive Availability of the target fluid is low and require heating and/or electrical excitation High saturability
^53^	Near-infrared transmission spectroscopy	Blood	No-extraction is required	NA	Claimed wearable but not flexible Complicated structure Non-invasive The target fluid is always available No-saturability Low precision
This work	Microwave resonator	ISF	No-extraction is required	Flexible ultra-thin dielectric substrate	Flexible and wearable structure Non-invasive The target fluid is always available No-saturability Lower precision

**Table 2:** Quantitative comparison between some of the state-of-the art microwave glucose sensors and the present design.

References	Microwave structure	Main frequency (GHz)	Sensitivity (converted to KHz/1 mM/l)	Distance between sample and the sensor (mm)	Sample liquid	Sample volume (µl)	Lowest glucose concentration measured (mM/l)	Comments
^41^	Discrete double split-ring Resonators	1.5	3.2	1	DI water	21,000	25	High power consumption Non-wearable and not flexible Readout circuitry should be attached to the sensor Low sensitivity
^54^	ENG-based microwave filter device	2	252	0.1	DI water	2	111	High power consumption Non-wearable and not flexible Readout circuitry should be attached to the sensor High sensitivity due to very small distance between the sample and the sensor
^55^	Microstrip Antenna Driven Ring Resonator	2	16	0.0014	DI water	90	555	Enzyme based Not flexible Readout circuitry should be attached to the sensor Medium sensitivity Saturable sensor
^56^	Split ring resonator	1.5	468	0.1	DI Water	NA	55.5	Non-wearable and not flexible Readout circuitry should be attached to the sensor High sensitivity due to very small distance between the sample and the sensor
This work	Chipless tag split ring resonator	4	38	1.4	Mimicked ISF	200	5	Ideal for wearable sensors Zero power consumption for the tag sensor Distant communication capability High sensitivity

## Methods

### Sensor fabrication

The utilized sensor in this work includes two parts; sensing tag and reader, both fabricated with almost the same process. Top face of the resonator is first printed on a glossy paper using a high-resolution printer. Printed pattern then transferred on a substrate by applying of high temperature and uniform pressure. For the reader part, ROGERS 5880 PTFE composite substrate with εr = 2.2, tan(δ) = 0.0004, thickness = 0.787 mm. Although it mentioned “substrate-less”, for simplifying the lab-based fabrication process of tag, it is printed on a ROGERS RO4450F with εr = 3.52, tan(δ) = 0.0004, thickness = 101 µm.

### Sample preparation

The saline composition is designed to mimic ISF as close as possible. A high concentration 10× stock solution is subsequently diluted with DI water to achieve actual concentration in ISF. The pH of the resulting saline is then buffered to pH 7.4 with NaOH and/or HCl, a value that mimics the pH of ISF. The final concentrations of the prepared solution are summarized in Table 3.

**Table 3:** Concentration of the ingredients of the prepared saline in all the related samples^57^.

Material	Concentration mM/l	Material	Concentration mM/l
Na^+^	140	Ca^2+^	1.5
Cl^−^	105	Mg^2+^	1
HCO_3_^−^	25	HEPES (buffer)	5
K^+^	5	HPO_4_^2+^	1.2

DI water used for all the experiments was double distilled water with 18 MΩ/cm resistance. For dehydration testing, the saline compartments were the same and the percentage of diluting DI water was reduced according to the dehydration percentage. All the test saline contained 10% horse serum (SIGMAALDRICH).

Simulation in Fig. 1c are accomplished using the tissue dielectric properties of humans as summarized in Table 4^58^. Skin samples were obtained from mice that were sacrificed for other research projects. The skin then shaved and sealed inside a plastic bag with a very small amount of saline surrounding to prevent excessive drying of the skin. The dielectric constant of the mouse skin is about 35^59^ which is in a good agreement with human skin dielectric constant used in simulations.

**Table 4:** Dielectric properties and thicknesses of different layers for human tissues utilized in Fig. 1.

Layer	Relative permittivity	Loss tangent	Thickness (mm)
Skin	38.00	0.283	2
Fat	5.28	0.148	4
Muscle	52.73	0.242	30

### Instruments and setups

**Figure 11:**
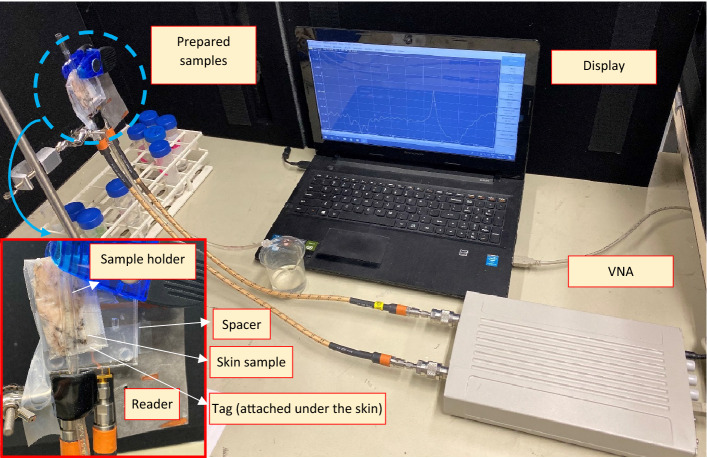
experimental setup including sensor structure, skin sample, holder and fixture, VNA and its interface S2 VNA Windows Software available at https://coppermountaintech.com/vna/s5085-compact-2-port-vna/.

All the microwave measurements have been accomplished using S5085 2-port vector network analyzer (VNA) from COPPERMOUNTAIN TECHNOLOGIES INC. The liquid samples were also tested inside a borosilicate tube with εr = 4.3 and tan(δ) = 0.0047 and wall thickness of 1 mm. The total exposed volume of liquid was 200 µl. Simulations have also been carried out using High Frequency Spectrum Simulator (HFSS). Figure 11 presents the fabricated sensor including the reader and the sensing tag as well as the experimental setup.

### Ethical approval

We confirm that all methods were carried out in accordance with relevant guidelines and regulations. We also confirm that all experimental protocols were approved by Research Ethics Office (REO) University of Alberta.

## Conclusion

Herein, we report the design and testing of a novel non-invasive wearable glucose monitoring sensor with zero power consumption and high sensitivity that is based on microwave planar resonator technology. The sensor is actually a metallic trace which could be taped over the skin. The impressive performance of the sensor, which removes many barriers against utilization of microwave resonator sensors for biomedical applications and especially wearable electronics, have been attained as the results of its improved design. Electromagnetic coupling between the reader and the tag, provides the distinct possibility of distant sensing and eliminating the requirement of a built-in power supply as the readout and communication circuitries can be integrated in the sensor’s reader unit. Moreover, substrate removal of the tag results in enhanced sensitivity of the sensor. This important improvement has been achieved as the result of making the permittivity of the MUT as the main defining parameter of the resonance frequency of the sensor. For testing the robustness of the sensor against variations of possible interferers in ISF, impacts of electrolytes variations as well as dehydration have been tested. Any electrolyte variation effects, over a physiological range of concentrations, resulted in a negligible frequency shift of the sensor. Also, the confounding effects of low to moderate dehydration was very low but became significant for severe dehydration. In summary, our results present a novel approach to the biosensing of physiological parameters such as glucose that represents a marked improvement over existing non-invasive sensor technologies.
